# Endometriosis, Angiogenesis and Tissue Factor

**DOI:** 10.6064/2012/306830

**Published:** 2012-07-11

**Authors:** Graciela Krikun

**Affiliations:** Department of Obstetrics, Gynecology & Reproductive Sciences, Yale University, 333 Cedar Street, New Haven, CT 06510, USA

## Abstract

Tissue factor (TF), is a cellular receptor that binds the factor VII/VIIa to initiate the blood coagulation cascade. In addition to its role as the initiator of the hemostatic cascade, TF is known to be involved in angiogenesis via intracellular signaling that utilizes the protease activated receptor-2 (PAR-2). We now review the physiologic expression of TF in the endometrium and its altered expression in multiple cell types derived from eutopic and ectopic endometrium from women with endometriosis compared with normal endometrium. Our findings suggest that TF might be an ideal target for therapeutic intervention in endometriosis. We have employed a novel immunoconjugate molecule known as Icon and were able to eradicate endometrial lesions in a mouse model of endometriosis without affecting fertility. These findings have major implications for potential treatment in humans.

## 1. Introduction

Normal ovarian functioning requires the coordinated activity of the hypothalamus, which secretes gonadotropin-releasing hormone (GnRH), the pituitary, which secretes the luteinizing hormone (LH) and follicle-stimulating hormone (FSH), the ovary, which secretes estrogens and progesterone, inhibins, activins, and other ovarian modulators [1]. The endometrium in turn responds to estrogen and progesterone [1]. Insulin-like growth factors (IGFs) and their binding proteins (IGFBPs) are important for endometrial development during the menstrual cycle and have mitogenic, differentiative, and antiapoptotic properties and participate in endometrial growth, differentiation, and apoptosis [2].

As a result, the human endometrium undergoes cyclic morphologic as well as molecular changes in preparation for receiving the incoming blastocyst and initiation of pregnancy [3]. If implantation does not occur, the endometrium is shed and menstruation occurs. These events involve extensive tissue remodeling, characterized by waves of endometrial cell proliferation, differentiation, recruitment of inflammatory cells, apoptosis, and tissue breakdown by metalloproteases, menstruation and ultimately regeneration [3]. The ability of ovarian hormones to trigger such diverse physiological responses is foremost dependent upon interaction of activated steroid receptors with specific transcription factors, such as Forkhead box class O (FOXO) proteins, involved in cell fate decisions [4], and HOX genes which encode evolutionarily conserved transcription factors essential to endometrial development, and endometrial receptivity [5]. Furthermore, microRNAs (miRNAs), small noncoding RNAs that function as posttranscriptional regulators of gene expression, have emerged as a major regulator system of steroid hormone responses in the female reproductive tract [4]. 

Lastly, the constant fluctuations in endometrial growth and shedding require that new vessels be developed. Indeed, the endometrium is one of the few tissues in the adult where physiological angiogenesis occurs [6]. Studies of endometrial angiogenesis are complicated by the continual changes in tissue growth and regression during the menstrual cycle and differences between the two different zones of the endometrium—the functionalis (the endometrial portion that sheds monthly) and basalis which appears to give rise to the new functionalis each month following menses. 

Several angiogenic factors have been identified and are believed to be involved in physiologic as well as pathologic angiogenesis in the human endometrium [7]. Vascular endothelial growth factor (VEGF) is considered to be the primary vasculogenic and angiogenic factor [7–9]. However, since the discovery of the angiopoietins [10] it has become clear that the angiogenic process is far more complex than previously thought. 

Indeed, tissue factor, the key initiator of the hemostatic cascade, is now also known to play a role in the process of angiogenesis [11, 12]. This subject will be discussed in more detail in the following.

## 2. Physiological Expression of Tissue Factor in the Endometrium

 Endometrial stromal cells from mid- to late secretory phase and decidual cells from gestational human endometrium display prominent immunohistochemical staining for tissue factor (TF). In contrast, no TF expression is observed during the proliferative phase [13, 14]. Consistent with the regulation by progesterone of the decidualization process *in vivo*, medroxyprogesterone acetate (MPA) or other synthetic progestins resulted in a significant induction of TF in primary stromal cell compared to basal levels. This increase paralleled the release of immunoreactive prolactin, a marker of decidualization [13, 14]. Northern analysis of RNA from cultured stromal cells indicated that MPA increased TF mRNA levels approximately 10-fold relative to control levels [13, 14]. In contrast, cultured stromal cell TF protein expression and mRNA levels were unaffected by exogenous estradiol added alone. However, we observed synergistic effect on TF expression after cells were primed with estrogen consistent with its function in elevating the stromal cell progesterone receptor [14]. These findings indicate that enhancement of endometrial stromal cell TF content is associated with progesterone induction of the decidualization process. In humans, trophoblastic invasion of the endometrial vasculature during blastocyst implantation risks hemorrhage. Therefore, increases in perivascular decidual cell tissue factor expression could serve to promote periimplantational endometrial hemostasis.

## 3. Endometriosis

Endometriosis is a gynecological disorder characterized by the presence of endometrial tissue outside of the uterus [15–17]. This disease affects up to 10% of all reproductive-aged women and 20%–50% of infertile women [15–18]. Despite its frequency and its impact on quality of life, our understanding of the pathogenesis of endometriosis remains incomplete. Endometrial lesions are primarily located on the pelvic peritoneum and ovaries but can also be found in the pericardium, pleura, lung parenchyma, and rarely the brain [19–21]. Implants can result in substantial morbidity, including pelvic adhesions and pain, allergies, fatigue, bowel problems, and infertility requiring extensive and often ineffective medical and surgical treatments [15–17, 22, 23]. Hence this disease is costly and both physically and psychologically debilitating. 

The etiology has been ascribed to retrograde menstruation, coelomic metaplasia, cells hematogenic and lymphogenic spread, remnants of the Mullerian duct, and endometrial stem/progenitor cells [15–17, 24, 25]. However, it also involves a complex interplay of genetic, anatomic, environmental, and immunologic factors [26–29]. Intense macrophage infiltration and excess cytokine expression play critical roles in the development of endometriosis-related chronic inflammatory processes [18, 30–34]. Endometriotic implant nidation also requires remodeling of the local peritoneal environment mediated by extracellular matrix (ECM) degrading proteases [35]. Matrix metalloproteinases (MMPs) play the dominant role in such tissue remodeling. Endometriotic lesions display enhanced expression of MMP-1, -3, and -7 [35–37]. 

Many of these same findings have been ascertained in a baboon model of endometriosis [38, 39]. While the molecular mechanisms by which endometriotic lesions persist are not clear, the establishment and progression of endometriosis require angiogenesis providing a target for new therapies. 

## 4. Angiogenesis

While angiogenesis occurs throughout fetal growth and development, in adults it is limited to the endometrium during the menstrual cycle, the ovary during formation of the corpus luteum and in pathological states including wound healing, diabetic retinopathy, tumor growth, and endometriosis [40–44]. This process requires new endothelial cells to sprout and then recruit pericytes to form capillaries or smooth muscle cells to form larger vessels [43, 45]. Subsequent steps include focal ECM degradation, endothelial cell proliferation and migration, organization of endothelial cells into capillary networks and lumen formation [46–50]. Several factors are involved in physiologic as well as pathologic angiogenesis in the human endometrium [6, 20, 51–53]. 

Vascular endothelial growth factor (VEGF) is the primary vasculogenic and angiogenic factor [46–50, 54]. The VEGF gene contains 8 exons, 7 introns, and a 14 kb coding region. Alternative exon splicing of a single VEGF gene produces 6 different isoforms. These include the predominant isoform VEGF165, as well as VEGF121, VEGF145, VEGF183, VEGF189, and VEGF 206 [55, 56]. The prominent VEGF isoforms are expressed by the endometrium and by primary cultured endometrial stromal cells [6, 55–60]. Binding of VEGF to the Flt-1 and KDR surface receptors activates their tyrosine kinase function resulting in enhanced endothelial cell proliferation, migration, vascular permeability, and protease activity [55, 61].

While VEGF has been considered central to the process of endometrial angiogenesis, we now know that many other factors are critical as well. For example, the angiopoietins also play a crucial role by promoting pericyte recruitment and vascular branching [62–65]. To date, four members of the angiopoietin family have been discovered. The angiopoietins are glycosylated: secreted proteins, which have characteristic protein structures that contain coiled-coil domains in the N-terminal portion and fibrinogen-like domains in the C-terminal portion of the molecule 27, 28. The angiopoietin1/Tie2 receptor system appears to be involved in the secondary stages of blood vessel formation [46–50, 54]. 

Additionally, fibroblast growth factors (FGFs), platelet derived growth factors (PDGF), and interleukin-8 (IL-8) exert more complex effects on angiogenesis [66–70] making this process extremely intricate.

Lastly, TF has now been demonstrated to be involved in angiogenesis [6, 58–60] making it an ideal target in pathologic events. This is discussed in the following.

## 5. Endometriosis and Angiogenesis

 Endometriotic implants require neovascularization to survive, grow, and invade ectopic sites [54, 71–76]. While there are conflicting reports concerning the origin of endometriosis, there is general agreement that endometriosis is associated with a local inflammatory response and that vascularization at the site of invasion plays a decisive role in the pathogenesis of the disease. Just as observed in tumor growth, angiogenesis of the endometriotic cells in implanted places appears to be essential for endometriotic cells survival and development. It has been shown that endometriotic lesions are highly vascularized, and it is now widely accepted that the formation of new blood vessels in implanted places plays a key role in the growth of endometriotic cells [77]. The angiogenic dynamics may provide blood supply to the refluxed menstrual debris, enabling attachment, implantation, and growth of endometrial cells on ectopic places. Indeed, several proangiogenic factors and their corresponding receptors have been found in peritoneal fluid and endometrial tissues from women with endometriosis [77]. As a result, peritoneal fluid from women with endometriosis is highly angiogenic [71–76, 78]. Moreover, specific VEGF blockers suppress the growth of ectopic endometrial explants in a nude mouse model of endometriosis [61, 79, 80]. However, many of these inhibitors have serious untoward effects which may be acceptable in cases such as progressive cancers [81] but would not be advisable for endometriosis, a painful but benign disease. 

In addition to the classic angiogenic agents described previously, tissue factor (TF) can mediate angiogenesis using a variety of distinct intracellular signaling pathways, and TF is aberrantly expressed in pathologically growing endothelium [45, 82–86]. Our initial studies suggested that overexpression of TF may be integral for the growth and survival of endometriotic lesions [45]. 

## 6. The Role of Macrophages in Endometriosis

Despite the ubiquitous occurrence of retrograde menstruation, most women do not develop endometriosis. In susceptible women, a cytokine-rich peritoneal milieu promotes survival of endometriotic implants [77]. Affected patients display elevated peritoneal concentrations of  IL-1*β*, IL-6, IL-8, IL-10, and TNF*α*, as well as the potent macrophage (M*φ*) chemoattractants: macrophage chemoattractant protein-1 (MCP-1), RANTES, eotaxin, and IL-8 [87, 88]. Implants are also a rich source of M*φ* and their recruiting chemokines [88–92]. In turn, activated M*φ* also produce proinflammatory mediators, including TNF*α*, IL-1*α*, IL-1*β*, IL-6, IL-8, leukemia inhibitory factor (LIF), interferon-*γ* (IFN-*γ*), and MCP-1 [87, 88, 90–92]. These cytokines are known to induce TF in endothelial cells and/or recruit and activate monocytes/M*φ* *in vitro* [93, 94]. Since TF/VIIa/PAR-2 interactions induce cytokine expression [95, 96], we hypothesize the existence of a positive feedback loop whereby the induction of  TF perpetuates inflammation. In support of this hypothesis, our studies showed that endometriotic implants display intense TF and PAR-2 expression in M*φ*. Thus targeting cells overexpressing TF provides an innovative approach to the treatment of this disease [97]. 

## 7. Tissue Factor

TF is a cell membrane-bound glycoprotein (MW 46 kDa) comprised of a hydrophilic extracellular domain, a membrane-spanning hydrophobic domain, and a cytoplasmic tail of 21 residues [98, 99]. Biological activity of the mature protein requires posttranslational modification to include carbohydrate moieties [100–102]. Endothelial cells and other cells in contact with the circulation do not normally express TF. However, following vascular disruption, perivascular cell-bound TF binds to circulating factor VIIa to mediate the activation of both factor IX and X and ultimately to generate thrombin [98, 99, 103]. Tissue factor is expressed in the mesenchymal and epithelial cells of diverse tissues [104–106]. Our laboratory established that in normal endometrium, TF expression is limited to stromal cells of the secretory phase with far lower expression in glandular epithelium [13, 14, 107]. Specifically, we demonstrated that progesterone (P4) enhances endometrial stromal cell TF mRNA and protein expression *in vitro* and that immunohistochemical staining for TF protein and *in situ *hybridization signaling for TF mRNA were greatest in stromal cells from the P4-dominated secretory phase [13, 14]. In contrast, previous studies from our laboratory indicate that in endometriosis, TF is greatly overexpressed in both glandular epithelium and stromal cells irrespective of menstrual phase [45, 108]. Unexpectedly, the most intense TF immunostaining was observed in macrophages infiltrating endometriotic tissues. Even more unusual was the presence of immunoreactive TF in endometriotic endothelial cells [45, 109].

 In addition to its role in hemostasis, TF/VIIa binding has important coagulation-independent functions, especially in embryonic and oncogenic angiogenesis, leukocyte diapedesis, and inflammation [110]. Indeed, TF deficiency causes embryonic lethality in the mouse. Thus, TF^−/−^ null embryos die at embryonic day E10.5 and display disorganization of the yolk sac vasculature suggesting that TF plays a pivotal role in vasculogenesis [11, 111, 112]. The absence of reports of TF deficiency in humans suggests a parallel obligatory requirement. While TF/VIIa signaling plays a critical role in angiogenesis, the underlying molecular mechanisms remain controversial. For example, the requirement for concomitant factor Xa binding and the need for phosphorylation as well as the obligatory role of the TF cytoplasmic tail have all been debated [113]. Several recent studies indicate that type-2 protease-activated receptor**(**PAR-2) is intimately involved in TF-mediated angiogenesis [113–116].

## 8. Protease-Activated Receptor-2 (PAR-2)

 The PAR family consists of four distinct transmembrane G-protein-coupled receptors, with each member playing an important role in inflammation [117]. PAR ligand/agonists are serine proteases that bind to each receptor and then cleave its extended, extracellular N-terminus at a specific site within the protein chain to expose an N-terminal tethered ligand domain [117]. The latter then binds to and activates the cleaved receptor. PARs are “single use” receptors since proteolytic activation is irreversible, and the cleaved receptors are degraded by lysosomes [117]. Thrombin is the primary agonist for PAR-1, the prototypical family member [118]. However thrombin can also activate PAR-3 and PAR-4, whereas PAR-2 is primarily a receptor for trypsin and the trypsin-like enzymes, factors Xa and VIIa [118–122]. Synthetic peptides that mimic the tethered ligand for each receptor activate PARs without the requirement for proteolytic activity [123–125]. These synthetic agonists permit identification of the independent role of individual PARs in a given biologic function. For example, the PAR-2 agonistic peptide (PAR2AP), is specific for PAR-2 but does not activate PAR-1, -3, or -4 [123–125]. A recent report by Shi et al. [126] revealing that the tethered ligand formed by thrombin activated PAR-1 can activate PAR 2 suggests the existence of an unexpected interaction between these two receptors.

In the endometrium, Hirota et al. showed that expression of PAR-2 mRNA is increased between the late secretory and menstrual phases [127, 128]. This group has also shown that activation of PAR-2 enhances IL-8 and MMP-7 production in both endometrial epithelial cells and stromal cells [127, 128]. Mitogen-activated protein kinases (MAPKs) mediate PAR-2-dependent IL-8 secretion [129]. Additionally, inflammatory agents such as tumor necrosis factor-*α* (TNF*α*), IL-1*β*, and lipopolysaccharide (LPS) increase PAR-2 expression in human endothelial cells [130]. Given the potent neutrophils chemotactic and angiogenic effects of IL-8 and its interaction with MMPs to foster endometrial remodeling, PAR-2 activation likely plays a critical role in pathological states in the endometrium [45].

## 9. TF/VIIa Signaling through PAR-2

 The complex of TF/VIIa with or without factor Xa bound to PAR-2 promotes angiogenesis directly by inducing release of VEGF in multiple cell types via MAPK activation [131]. The TF/VIIa/PAR-2 complex also promotes angiogenesis, inflammation, and tumor cell migration, and invasion and these processes can be blocked by addition of site-inactivated VIIa, as well as by specific antibodies against TF [131]. However, TF/VIIa can also indirectly stimulate angiogenesis by the generation of thrombin [132, 133]. Binding of PAR-1 by thrombin also activates MAPK to enhance VEGF expression [134–136]. Taken together, these data support the hypothesis that TF-VIIa-PAR-2 cell signaling plays a central role in the survival of pathologic cellular growth such as carcinomas as well as more benign conditions such as endometriosis. Hence, blocking TF or PAR-2 may provide another pathway by which to inhibit aberrant angiogenesis.

## 10. Icon Abolishes Endometriotic Lesions in a Mouse Model of Human Endometriosis

 Icon is a novel chimeric antibody-like immunoconjugate molecule (Icon). Icon is composed of a mutated noncoagulation-inducing factor VII (fVII) domain targeting TF and an IgG1 Fc (fVII/IgG1 Fc) effector domain that activates an NK cell cytolytic response against the TF-bearing cells [137–140]. We have shown that Icon eradicates preestablished human endometriotic lesions in an athymic (ATN) mouse model without untoward systemic effects, altered fertility, or subsequent teratogenesis [109]. Specifically, Icon protein was synthesized by stable transfection of Chinese hamster ovary (CHO) cells as previously described [137–139]. To prevent the induction of coagulation following binding of Icon to TF, an amino acid substitution (Lysine 341 to Alanine) was introduced into the Icon fVII domain to block initiation of coagulation without reducing the molecule's strong affinity for TF [137]. The ATN mice were injected with approximately 1.0 mL packed endometrial tissue as previously described [109]. Upon gross morphological examination, 11 out of 15 treated mice had no sign of disease after 4 weekly 10 *μ*g Icon treatment compared to 5 out of 12 mice treated with a 5 *μ*g dose. By contrast, 12 out of 13 mice treated with vehicle control had well-defined endometrial lesions. For those animals treated with Icon and displaying residual lesions, the latter were significantly smaller than those found in control animals. Moreover residual lesions following treatment appeared to be avascular. 

Most importantly, unlike antiangiogenic treatments that can only target developing angiogenesis, Icon eliminates preexisting pathological vessels without untoward systemic effects, altered fertility, or subsequent teratogenesis [108].

## 11. Icon Targets Tissue Factor in Uterine Serous Papillary Carcinoma

Pathological angiogenesis, the formation of new capillary blood vessels from existing blood vessels into diseased tissues, has been previously reported to occur more frequently in endometrial carcinomas developing against a background of endometrial atrophy rather than carcinomas arising from a hyperplastic endometrium [141]. Tissue factor is aberrantly expressed in human cancers and on endothelial cells within the tumor vasculature [142–144]. Importantly, tumor cells characterized by a high production of TF and vascular endothelial growth factor are known to generate solid tumors characterized by intense vascularity and highly aggressive behavior [145, 146]. Consistent with this view, vascular endothelial growth factor expression at the invading tumor front is reported to be 4–10 times higher than in the inner tumor areas and is significantly associated with poor prognosis, particularly with advanced stage endometrial cancer [141]. 

In a recent study, we evaluated for the first time the *in vitro* potential of Icon as a novel immunotherapeutic agent against biologically aggressive uterine serous tumors (USPC) [147]. Cytoplasmic and/or membrane TF expression was observed in all 16 (100%). USPC samples were tested by immunohistochemistry. High expression of TF was found in 50% (three out of six) of the USPC cell lines tested by real-time PCR and flow cytometry when compared with normal endometrial cells. Uterine serous papillary adenocarcinoma cell lines overexpressing TF were highly sensitive to Icon and demonstrated that this molecule induced strong cytotoxicity against primary chemotherapy-resistant USPC cell lines overexpressing TF. Lastly, it has been demonstrated that Icon could separately induce murine natural killer (NK) cells and activate complement to kill cancer cells *in vitro* via antibody-dependent cell-mediated cytotoxicity (ADCC) and complement-dependent cytotoxicity (CDC) [148].

## 12. Discussion

 Prior studies from our laboratory have shown that in normal endometrium, progestins markedly enhance TF protein and mRNA expression in decidualized stromal cells during the luteal phase [13, 14, 149]. We have also shown that glandular epithelial cells display minimal TF expression throughout the menstrual cycle [13, 14, 149]. Upon discovery of aberrant endothelial TF expression in the endometriotic neovascularture we evaluated whether a novel, immunotherapy targeting endothelial TF could eradicate endometriotic implants. Increased expression of TF  in ectopic endometrium from patients with endometriosis compared with controls is a novel finding. It may reflect the known association of endometriosis with increased inflammatory cytokine production [73, 150, 151]. It is well established that IL-1 *β* and tumor necrosis factor-*α* acting via the NF*κ*B transcription factor increase TF gene expression in endothelial and other cells types [152]. Increased TF expression in endometrial endothelial cells may also reflect genetic polymorphisms in the promoter region of genes known to regulate TF expression as well as the TF promoter region itself [153–155]. 

The aberrant expression of TF in ectopic endometrial endothelium suggested that TF might be an ideal therapeutic target for endometriosis. Towards this end, we employed the immunoconjugate molecule, Icon, in a mouse model of endometriosis. Previous studies have demonstrated that Icon targeted TF anomalously expressed on the vasculature of malignant tumors as well as on parenchymal cells within solid tumors [137–139]. Icon also reduced the formation of pathological choroidal neovasculature which is associated with macular degeneration [156, 157]. Thus, Icon takes advantage of endothelial TF expression in pathological neovasculature while having no effects on normal vessels. Icon therapy was shown to eradicate endometriotic lesions in a mouse model of endometriosis [108]. 

Importantly, because Icon targets not only neo-angiogenesis, but aberrant preexisting vessels, it is the only available compound that could be potentially used to successfully treat preexistent, vascularized lesions. Indeed, women suffering from endometriosis are typically not diagnosed for several years [158, 159] and, thus, would already have established lesions. Although several treatments for endometriosis are available, they are associated with high recurrence rates and considerable side effects [80]. Prior studies have confirmed that Icon treatment does not produce toxicity in various animal strains [137, 139, 156, 157, 160], and we confirm no untoward effects on adult mice treated with Icon. In addition, we now report that treatment does not interfere with subsequent fertility nor does it give rise to teratogenic effects. Hence, Icon may be an ideal drug of choice in the treatment of endometriosis and in particular for reproductive-aged women suffering with this disease who desire subsequent fertility. 
